# P53 and Ki-67 as prognostic markers in triple-negative breast cancer patients

**DOI:** 10.1371/journal.pone.0172324

**Published:** 2017-02-24

**Authors:** Yunbao Pan, Yufen Yuan, Guoshi Liu, Yongchang Wei

**Affiliations:** 1 Department of Laboratory Medicine, Zhongnan Hospital of Wuhan University, Wuhan, Hubei, China; 2 Department of Pathology, Anyang Tumor Hospital, Anyang, Henan, China; 3 Department of orthopedics, the Second Affiliated Hospital of Nanchang University, Nanchang, Jiangxi, China; 4 Department of Radiation and Medical Oncology, Zhongnan Hospital of Wuhan University, Wuhan, Hubei, China; University of North Carolina at Chapel Hill School of Medicine, UNITED STATES

## Abstract

Triple-negative breast cancer (TNBC) is an aggressive subgroup of breast cancer lack of effective target therapy. This study was to investigate the prognostic role of p53 and Ki-67 in 156 cases of TNBC patients. Logistic regression analysis was used to examine the association between clinical parameters and recurrence. Univariate and multivariate analyses were used to examine the association between clinical characteristics and disease-free survival (DFS) or overall survival (OS). Survival analyses using the Kaplan-Meier method were performed to examine the association between p53/Ki-67 and DFS and OS. Our data showed that p53 was positive in 71.3% and the Ki-67 high index was in 82.8% of TNBC. Elevated p53 and Ki-67 were associated with histological grade. The tumor size, lymph node involvement, and p53 expression are associated with risk of recurrence. Tumor size, lymph node involvement, family history, Ki-67 and p53 are independent variables associated with either DFS or OS. TNBC patients with positive p53 or Ki-67 high index or family history of cancer have a significant association with worse prognosis. This study suggests that p53, Ki-67 and family history are useful prognostic markers in TNBC.

## Introduction

Breast cancer is the most prevalent cancer and the leading cause of cancer death in female worldwide. It also accounts for 25% of all cancer cases and 15% of all cancer deaths in women [1]. Triple-negative breast cancer (TNBC) takes up about 15% of all breast cancers and lacks estrogen receptor and progesterone receptor expression as well as human epidermal growth factor receptor 2 (HER2) amplification. TNBC doesn’t benefit from endocrine therapy or targeted therapy in contrast with the other subgroups [2, 3]. Compared to other subtypes of breast cancer, TNBC is more biologically aggressive and has higher recurrence rate, higher frequency of metastasis and worse survival [4, 5]. The clinicopathological parameters of this subgroup consist of large tumors size, multiple apoptotic cells, high proliferative index, highly undifferentiated, central necrosis and high positivity of lymph node involvement. The major histological type of TNBC is ductal and less commonly the medullary [6].

The number of cancer-related parameters available to predict the prognosis of breast cancer patients has grown considerably in recent years. Prognostic factors of breast cancer include histological features (histological type, histological grade, lymphovascular invasion), tumor size, lymph node status, steroid hormone receptors status and age [7–9]. Prognostic and predictive biomarkers, including p53 [10] and Ki-67 [11], were also identified in breast cancer. *P53* (also known as TP53) locates on chromosome 17p13 and encodes p53 transcription factor. P53 plays a vital role in determining cell fate exposed to DNA damage stimuli [12]. Alterations of p53 have been investigated with particular interest in the recent years. Studies suggest that *P53* gene is the most frequently mutated tumor suppressor gene in human malignancy [13], and 30% breast cancers have *P53* mutation. The frequency of *P53* mutation in breast cancer relies on molecular subset, luminal subgroup has lowest mutation and basal subgroup has highest mutation [14]. The mutation of *P53* gene may represents an early event in tumor progress, because it is evident at the in situ phase of cancer growth. Additionally, *P53* mutation probably stimulates cell proliferation and renders aggressive phenotype. The availability of detecting mutant p53 protein on formalin-fixed paraffin-embedded (FFPE) tissue has allowed the retrospective studies of patients with a long follow-up.

Ki-67 is a non-histone nuclear protein and correlated with cell growth. Ki-67 expression varies through cell cycle, with different expression levels in G1, G2/M, and S phases but undetectable in G0 phase. Ki-67 associates with cell cycle progress and the short half-life confer it an effective biomarker for assessing growth fraction of tumor cells. Ki-67 is one of the most widely used immunohistochemistry (IHC) proliferation antigen and has been confirmed as an independent predictive and prognostic factor in breast cancer [15, 16]. Ki-67 is an important parameter in sub-classifying luminal tumors into a good prognosis luminal A subtype and a worse prognosis luminal B subtype [17]. While the prognostic value of Ki-67 in TNBC remains to be determined.

In the current study, we investigated the association between p53, Ki-67, clinical characteristics, family history of cancer, and recurrence, DFS and OS in TNBC patients.

## Materials and methods

### Patients and methods

One hundred and fifty-six TNBC patients treated at Anyang Tumor Hospital from August 2010 to December 2013 were included in this study. All of medical records were reviewed retrospectively. The inclusion criteria for all participants were: aged 18 years; diagnosis of TNBC. Exclusion criteria were: preoperative chemotherapy or radiotherapy; deficiency of clinical data or lack of follow up. All patients were diagnosed TNBC because both estrogen and progesterone receptor were 0% by IHC, and HER2 was 0% by IHC or 1+ and 2+ score without gene amplification confirmed by FISH. FFPE tumor samples were selected for IHC staining with primary antibody against p53 and Ki-67. Tissue was defined as p53 positive if any cancer cells positively stained. We considered a high Ki-67 index ≥14% cell staining [18]. This study was approved by Anyang Tumor Hospital Ethics and Scientific Committee. Inquiries about the date and mode of death were made by directly corresponding with the referring physician and/or the family of the deceased patient, with written permission obtained at the time of undertaking surgery from all patients and/or their relatives, allowing the use of personal data for research purposes.

### Follow-up and statistical snalysis

Breast cancer recurrence was defined as the regional or distant relapse in any site [19]. Disease-free survival (DFS) was calculated as the time from initial diagnosis to recurrence, metastasis or death attributable to any cause. Overall survival (OS) was defined as the period from initial diagnosis to death regardless of breast cancer related or not. The median follow-up time among the 156 patients was 48 months, ranging from 4 months to 69 months. Before closing the research database for analysis in June 2016, the authors updated the follow-up data of patients who had not visited our outpatient department for more than three months. Patient follow-up was censored at the time of death or finalization of the study. 18 patients who were lost to follow-up have been ruled out the studies.

Comparisons between groups were performed using Chi-square test for categorical variables. Comparisons of the percent of p53- or Ki-67-positve cells among the three subgroups of patients were tested by one-way ANOVA and LSD tests. Associations between P53 and Ki-67 expression and histological grade were determined using Pearson correlation. Logistic regression was used in multivariate analyses to identify risk factors impacting recurrence. Survival curves were plotted using the Kaplan-Meier method and differences between the survival curves were determined using the log-rank test. A p value of <0.05 was considered significant. The calculations were performed using SPSS version 22.0 software (SPSS, Chicago, IL).

## Results

### Patient characteristics

**Fig 1:**
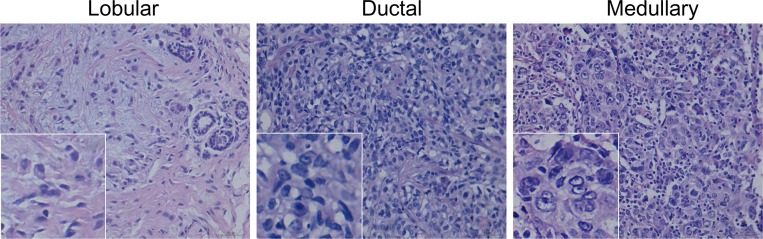
HE staining of TNBC tissue. (A) Lobular cancer. (B) Ductal cancer. (C)Medullary cancer.

In the present study, patients’ median age was 51 years (range 28–85). 78 cases (50%) were menopause. The main histological type was lobular in 11 (7.1%), ductal in 141 (90.4%), medullary in 4 (2.5%) (Fig 1). 19 (12.2%) had a grade 1 tumor; 89 (57.1%) had a grade 2 tumor; 48 (30.8%) had a grade 3 tumor. Based on tumor staging system, most patients were defined as stage II (88, 56.4%) and stage I (36, 23.1%). 66 patients (42.3%) had lymph node involvement. 4 patients (2.6%) developed distant metastasis. The patients’ clinicopathologic characteristics were summarized in Table 1.

**Table 1:** Characteristics of the Patients (n = 156).

Variable	Number (%)
Age	
<60	123 (79%)
≥60	33 (21%)
Histological type	
Lobular	11 (7.1%)
Ductal	141 (90.4%)
Medullary	4 (2.5%)
Stage	
I	36 (23.1%)
II	88 (56.4%)
III	28 (17.9%)
IV	4 (2.6%)
Grade	
G1	19 (12.2%)
G2	89 (57.1%)
G3	48 (30.8%)
Tumor size	
T1	56 (35.9%)
T2	85 (54.5%)
T3&T4	15 (9.6%)
Lymph node metastasis	
Yes	66 (42.3%)
No	90 (57.7%)
Distant metastasis	
No	152 (97.4%)
Yes	4 (2.6%)
Ki-67	
<14%	26 (16.7%)
≥14%	130 (83.3%)
P53	
Negative	42 (26.9%)
Positive	114 (73.1%)
Family history	
Yes	50 (32.1%)
No	106 (67.9%)
Menopause	
Yes	78 (50%)
No	78 (50%)

### Association between p53/Ki-67 and clinicopathological parameters

**Fig 2:**
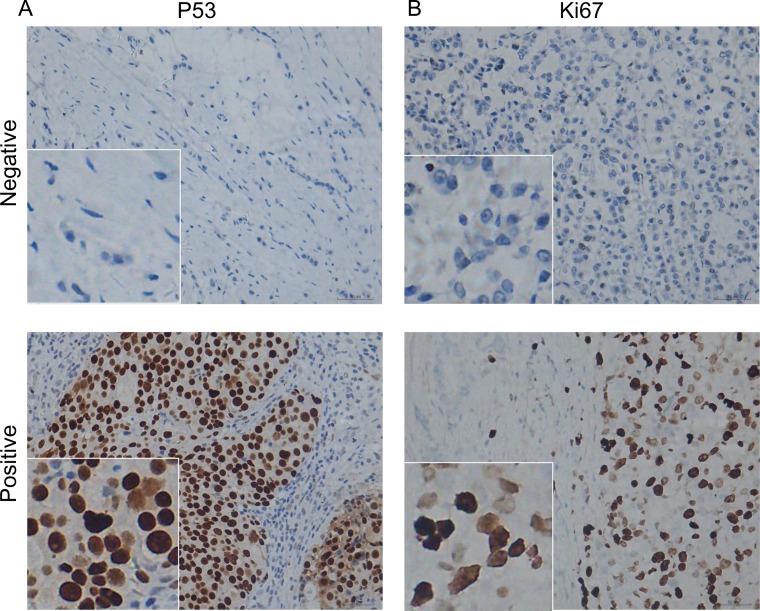
Immunohistochemical analysis of p53 and Ki-67 in TNBC. (A) Negative and positive p53 staining. (B) Negative and positive Ki-67 staining.

**Fig 3:**
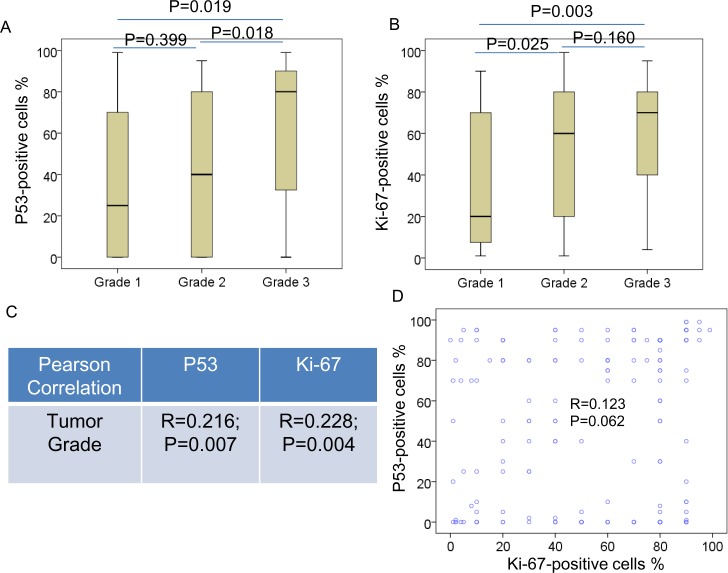
P53 and Ki-67 associates with tumor grade in TNBC. Box plots indicating the distribution of the p53 (A) and Ki-67 (B) and tumor grade, P values were from one-way ANOVA and LSD tests. (C) P53 and Ki-67 was associated with tumor grade in TNBC. (D) P53 was associated with Ki-67 in TNBC. The R and P values were from Pearson Correlation.

The expression of p53 and Ki-67 in TNBC were detected by IHC (Fig 2). Correlations between p53 positive cases or Ki-67 high index cases and clinicopathological parameters were summarized in Table 2. The distribution of most clinicopathological parameters is similar in the p53 positive patients, as well as in the patients with Ki-67 high index. However, most patients with high Ki-67 expression aged <60 years (<60 *vs*. ≥60, 82.3% vs. 17.7%, p = 0.018), and most patients with high Ki-67 expression were in grade 2 (G1 *vs*. G2 and G3, 9.2% *vs*. 56.9% and 33.8%, respectively, p = 0.019) (Table 2). In addition, the data indicated that the percentage of p53-positive and Ki-67-positive cells in cancer with higher tumor grade was much higher than those with grade 1 (Fig 3A and 3B). Elevated p53 and Ki-67 levels were correlated with tumor grade (Fig 3C). In addition, p53 levels was associated with Ki-67 levels in TNBC (Fig 3D). However, we did not observe any association between p53/Ki-67 and histological type, tumor stage, family history nor menopause.

**Table 2:** Expression of p53 positivity/Ki-67 high index in each characteristic.

Variable	Cases of P53 (+) (n = 115)	P	Cases of Ki-67 (high) (n = 130)	P
Age				
<60	92 (80%)	χ^2^ = 0.349	107 (82.3%)	χ^2^ = 5.604
≥60	23 (20%)	p = 0.555	23 (17.7%)	p = 0.018
Histological type				
Lobular	7 (6.1%)	χ^2^ = 0.621	7 (5.4%)	χ^2^ = 3.592
Ductal	105 (91.3%)	p = 0.733	120 (92.3%)	p = 0.166
Medullary	3 (2.6%)		3 (2.3%)	
Stage				
I	29 (25.2%)	χ^2^ = 2.997	27 (20.8%)	χ^2^ = 3.210
II	63 (54.8%)	p = 0.392	76 (58.5%)	p = 0.360
III	19 (16.5%)		23 (17.7%)	
IV	4 (3.5%)		4 (3.1%)	
Grade				
G1	13 (11.3%)	χ^2^ = 1.161	12 (9.2%)	χ^2^ = 7.971
G2	64 (55.7%)	p = 0.560	74 (56.9%)	p = 0.019
G3	38 (33.0%)		44 (33.8%)	
Tumor size				
T1	42 (36.5%)	χ^2^ = 1.613	43 (33.1%)	χ^2^ = 3.319
T2	64 (55.7%)	p = 0.446	75 (57.7%)	p = 0.190
T3&T4	9 (7.8%)		12 (9.2%)	
Lymph node metastasis				
Yes	47 (40.9%)	χ^2^ = 0.371	56 (43.1%)	χ^2^ = 0.189
No	68 (59.1%)	p = 0.543	74 (56.9%)	p = 0.664
Distant metastasis				
No	111 (96.5%)	χ^2^ = 1.464	126 (96.9%)	χ^2^ = 0.821
Yes	4 (3.5%)	p = 0.226	4 (3.1%)	p = 0.365
Ki-67				
<14%	20 (17.4%)	χ^2^ = 0.165		
≥14%	95 (82.6%)	p = 0.684	130 (100%)	n.a.
P53				
Negative			35 (26.9%)	χ^2^ = 0.165
Positive	115 (100%)	n.a.	95 (73.1%)	p = 0.684
Family history				
Yes	40 (34.8%)	χ^2^ = 1.499	41 (31.5%)	χ^2^ = 0.094
No	75 (65.2%)	p = 0.221	89 (68.5%)	p = 0.759
Menopause				
Yes	57 (49.6%)	χ^2^ = 0.033	64 (49.2%)	χ^2^ = 0.185
No	58 (50.4%)	p = 0.856	66 (50.8%)	p = 0.667

n.a. = not applicable.

### Survival analysis

**Fig 4:**
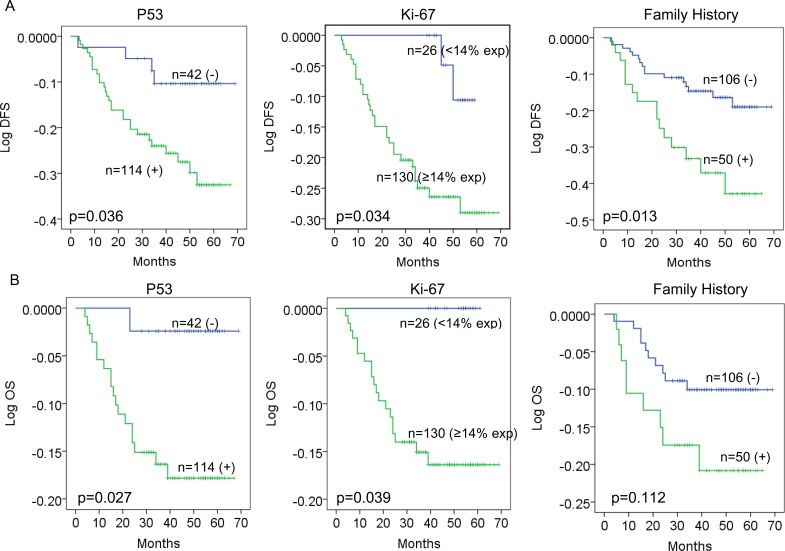
P53, Ki-67 and family history predict survival in TNBC. Estimated disease-free survival (DFS) (A) and overall survival (OS) (B) curves for p53, Ki-67 and family history.

The median follow-up time was 48 months (range 3–69 (DFS) and 4–69 (OS)). In order to evaluate the prognostic influence of p53/Ki-67 expression, we carried out Kaplan-Meier analyses to compare grouped patients. The survival curves demonstrated that patients with positive p53 or Ki-67 high index had a significant association with worse DFS and OS (DFS, p = 0.036, p = 0.034; OS, p = 0.027, p = 0.039; Fig 4A and 4B). Although family history did not have a significant influence on OS (p = 0.112; Fig 4B), patients with family history of cancer tended to have worse DFS (p = 0.013; Fig 4A).

### Prognostic factors

Logistic regression was used in multivariate analyses to identify risk factors impacting recurrence. Variables assessed in multivariate analysis and significant variables were shown in Table 3. In the logistic regression, tumor size, lymph node metastasis and p53 positivity were associated with recurrence with a more prominent predictive effect (P < 0.05) (Table 3). P53/Ki-67 levels must be divided into categorical variable (positive *vs*. negative, high index *vs*. low index and negative) in our logistic regression and Cox regression models.

**Table 3:** Logistic regression analysis of factors predicting recurrence.

Parameters	B	S.E.	Wald	df	Sig.	Exp (B)	95% CI
Age (≥60y)	0.373	0.557	0.448	1	0.503	1.452	0.487–4.329
Menopause (+)	-0.118	0.516	0.052	1	0.820	0.889	0.324–2.443
Histological type			0.153	2	0.926		
Lobular	-0.338	0.944	0.128	1	0.720	0.713	0.112–4.536
Ductal	0.175	1.263	0.019	1	0.890	1.192	0.100–14.177
Tumor size			5.680	2	0.058		
T1	0.849	0.536	2.516	1	0.113	2.338	0.819–6.679
T2	1.807	0.774	5.446	1	0.020	6.094	1.336–27.802
LN metastasis (+)	1.558	0.462	11.378	1	0.001	4.749	1.921–11.741
Family history (+)	0.874	0.451	3.762	1	0.052	2.396	0.991–5.795
Ki-67 (≥ 14%)	1.329	0.844	2.480	1	0.115	3.777	0.722–19.746
P53 (+)	1.439	0.633	5.171	1	0.023	4.218	1.220–14.585
Constant	-4.390	0.829	28.024	1	0.000	0.012	

LN: lymph node.

On univariate survival analysis, conventional prognostic parameters, including tumor size, lymph node metastasis, family history of cancer, and P53 positivity, reached significance for DFS (p<0.05 for all) (Table 4). In addition, tumor grade, tumor size, and lymph node metastasis were factors affecting OS of TNBC (Table 5).

**Table 4:** Univariate and multivariate survival analysis for disease-free survival.

	Univariate		Multivariate	
Parameters	P	HR (95% CI)	P	HR (95% CI)
Age (<60y vs. ≥60y)	0.952	1.03 (0.44–2.37)	0.466	1.39 (0.58–3.34)
Menopause (yes vs. no)	0.996	1.00 (0.50–2.00)	0.517	0.75 (0.33–1.71)
Histological type	0.979	1.01 (0.43–2.40)	0.839	0.91 (0.35–2.32)
Tumor grade(1 vs. 2 vs. 3)	0.220	1.42 (0.81–2.48)	0.891	0.96 (0.51–1.80)
Tumor size (T1 *vs*. T2 *vs*. T3)	0.006	2.15 (1.25–3.71)	0.005	2.42 (1.30–4.52)
LN metastasis (+ vs. -)	0.0004	4.03 (1.86–8.70)	0.001	3.57 (1.64–7.77)
Family history (+ vs. -)	0.016	2.35 (1.18–4.70)	0.048	2.03 (1.01–4.08)
Ki-67 (≥14% vs. <14%)	0.073	3.71 (0.89–15.57)	0.047	4.34 (1.02–18.52)
P53 (+ vs. -)	0.046	2.91 (1.02–8.31)	0.020	3.55 (1.23–10.30)

HR: hazard ratio; CI: confidence interval; LN: lymph node.

**Table 5:** Univariate and multivariate survival analysis for overall survival.

	Univariate		Multivariate	
Parameters	P	HR (95% CI)	P	HR (95% CI)
Age (<60y vs. ≥60y)	0.60	1.31 (0.47–3.65)	0.009	7.10 (1.64–30.86)
Menopause (yes vs. no)	0.70	1.22 (0.45–3.31)	0.055	0.30 (0.09–1.02)
Histological type	0.74	0.71 (0.094–5.31)	0.604	1.39 (0.40–4.79).
Tumor grade (1 vs. 2 vs. 3)	0.038	2.269 (1.05–4.93)	0.145	1.87 (0.81–4.35)
Tumor size (T1 vs. T2 vs. T3)	0.000	4.10 (1.98–8.48)	0.000	6.83 (2.61–17.88)
LN metastasis (+ vs. -)	0.001	7.93 (2.31–27.21)	0.004	6.34 (1.83–21.99)
Family history (+ vs. -)	0.120	2.04 (0.83–5.03)	0.024	3.19 (1.16–8.71)
Ki-67 (≥14% vs. <14%)	0.197	27.52 (0.179–4235)	0.966	1394147 (0.00–2.216E+290)
P53 (+ vs. -)	0.054	7.26 (0.97–54.43)	0.007	19.70 (2.23–174.03)

HR: hazard ratio; CI: confidence interval; LN: lymph node.

To evaluate whether p53 positivity and Ki-67 high index in TNBC were independent predictors of DFS and OS, a multivariate analysis was performed with the following variables: age, menopause status, histological type, tumor grade, tumor size, lymph node involvement, family history, Ki-67 and P53 expression. Age (p = 0.009, only for OS), tumor size (p = 0.005 and p<0.001 for DFS and OS, respectively), lymph node involvement (p = 0.001 and p = 0.004 for DFS and OS, respectively), family history (p = 0.048 and p = 0.024 for DFS and OS, respectively), Ki-67 high index (p = 0.047, only for DFS), and P53 (p = 0.020 and p = 0.007 for DFS and OS, respectively) were significant prognostic factors for TNBC (Tables 4 and 5). Multivariate analysis identified Ki-67 high index and P53 positivity as significant independent factors for poor DFS and OS in TNBC.

## Discussion

TNBC have poor prognosis attributed to the aggressive biology and deficiency of targeted agents [3]. Better understanding the biological behavior is urgent to improve patients’ outcomes. In the present study, we retrospectively analyzed 156 patients to investigate the association between p53/Ki-67 expression with clinical parameters and prognosis of TNBC. All the patients were from one hospital ensured the stability of the test quality of pathological biomarkers.

Although various methodological and clinical settings have been applied to explore p53 status for predicting therapy response and patients’ outcomes, results are contradictory [20]. Missense mutation of *P53* gene induces stable detectable mutant p53 protein, whereas truncating *P53* gene mutations yields unstable p53 proteins that cannot be detected by IHC [21, 22]. Besides, wildtype p53 may accumulate in cells resulted from DNA damage or binding to other proteins and thus show strong immunoreactivity [21, 23].

We examined p53 expression in 156 cases of TNBC and found it was positive in 71.3% of cases, which was in consistent with reported positivity rates of p53 expression (56% to 71%) in TNBC [22]. Although the association between p53 and clinical features varies in studies, we found that histologic grade was the only variable correlated with p53 expression. Furthermore, studies on the prognostic significance of p53 expression as evaluated by IHC showed contrary conclusions [21, 24, 25]. We found p53 positivity was correlated with worse prognosis in TNBC, which was in agreement with previous studies that p53 mutation has negative prognostic significance in breast cancer patients [26, 27].

The usage of Ki-67 as a prognostic marker in breast cancer has been widely studied, high Ki-67 expression has been demonstrated to be correlated with larger tumor size, higher histological grade, lymph node involvement, shorter DFS and OS in breast cancer [28, 29]. Moreover, it was reported a positive association between Ki-67 expression and tumor response to neoadjuvant chemotherapy [30, 31]. However, only a few groups have studied it in the triple negative subgroup [32–34]. Some studies [35] evaluated the prognostic value of Ki-67 in the whole cohort of breast cancer, but the number of cases in TNBC was quite small and this may limit the ability of Ki-67 to identify clinically distinct subtypes.

Though Ki-67 staining levels of 10%–20% have been the most common to dichotomize populations [36], it still lacks a standardized cut-off value in the clinical practice. It was reported that a high Ki-67 expression (≥10%) was significantly associated with poor relapse-free survival and overall survival in TNBC [33]. Ki-67 labeling index was associated with different prognosis subgroups in node-negative TNBC with a cut-off value of 35% [33]. In line with these results, our study found that high expression of Ki-67 (≥ 14%) is significantly correlated with a worse prognosis in TNBC patients.

Up to 20% breast cancer patients are believed to be hereditary and was distinguished by multiple cases of breast and/or other cancers among relatives [37]. Mutations in *BRCA1* and *BRCA2* genes plays vital role in the majority of hereditary cases of breast cancer [37]. The prevalence of family history of cancer among first-degree relatives in our study was 32.1%, which is close to the range (6.2%-27.1%) reported in the literatures [38, 39]. Our data also indicated patients with family history of cancer tended to associate with worse DFS.

## Conclusions

Taken together, immunohistochemical evaluation of p53 and Ki-67 proteins might stratify TNBC into subtype with different aggressiveness and prognosis.
